# Editorial: African Swine Fever in Smallholder and Traditional Pig Farming Systems: Research, Challenges and Solutions

**DOI:** 10.3389/fvets.2022.878928

**Published:** 2022-04-26

**Authors:** Mary-Louise Penrith, Klaus Depner, Ferran Jori, Michel Dione, Robyn Alders, Erika Chenais

**Affiliations:** ^1^Department of Veterinary Tropical Diseases, Faculty of Veterinary Science, University of Pretoria, Pretoria, South Africa; ^2^Institut für Internationale Tiergesundheit/One Health, Friedrich-Loeffler-Institute, Greifswald, Germany; ^3^UMR ASTRE (Animals, Health, Territories, Risk and Ecosystems), CIRAD-INRAE, Montpellier, France; ^4^Animal and Human Health Program, International Livestock Research Institute, Dakar, Senegal; ^5^Development Policy Centre, Australian National University, Canberra, ACT, Australia; ^6^Kyeema Foundation, Brisbane, QLD, Australia; ^7^Department of Disease Control and Epidemiology, National Veterinary Institute, Uppsala, Sweden

**Keywords:** African swine fever, control and prevention, smallholder pig farmers, socioeconomic impacts, participatory approaches

African swine fever (ASF) is a devastating disease of pigs that originated in south-eastern Africa in a sylvatic cycle involving warthogs and soft ticks of the *Ornithodoros moubata* complex. The disease is now global with presence in five continents (1–3). While it is greatly feared throughout all parts of the pig industry worldwide, its effects in the smallholder and traditional pig farming sectors that predominate in most low-income countries and are also present in many higher income countries are often underestimated. Smallholder pig farming is important for many reasons, providing crucial household income, improving nutrition and food security by supplying an affordable source of high quality protein and bioavailable micronutrients as well as fulfilling social and cultural obligations that keep ancient traditions alive and improve social coherence and sustainability (4, 5). Pig production units categorized as “backyard” and “village” are over-represented in reports of ASF outbreaks to the World Organization for Animal Health (OIE) from low- and middle-income countries, and they may be seen as a threat to the commercial pig industry and a hindrance to eradication of ASF. However, recognizing the importance of pigs to numerous smallholders and their potential contribution to poverty alleviation, preventing ASF in smallholder pigs has become a focus of attention and research.

Early detection and rapid reaction are important for efficient control of ASF, but are difficult to achieve in the smallholder sector as it involves large numbers of pig owners in rural areas with inadequate infrastructure and animal health service provision. Prevention of ASF outbreaks by improving biosecurity in production systems and the value chains that serve them is key to limit losses and can only be achieved by working with the pig owners to decide what is feasible for them, using participatory methods. Much has been done to create awareness of ASF and how to prevent it in the absence of an effective vaccine, but many of the recommendations are not feasible for people with limited resources, including inadequate access to finance (6).

This Research Topic is aimed at collecting articles that will add to our knowledge of the traditional and smallholder pig sectors, the impacts of ASF and challenges faced in these sectors, and the approaches that are being used to support feasible and effective prevention and control.

In this special Research Topic there are 12 articles addressing these aspects in seven countries across three continents. Seven articles focus on management of ASF, three on socio-economic impact, one on rapid field diagnosis and one on policy and legislation. The articles are briefly summarized according to those categories.

In the article by Mutua and Dione the epidemiological role of factors such as the context of pig value chains and human risk behaviors is reviewed with a focus on smallholder pig systems in Africa. In this regard farm level biosecurity is particularly emphasized, and factors influencing its adoption are highlighted in the article. Priority areas to consider while designing interventions to improve pig productivity are identified to be socio-cultural factors, weaknesses at the disease control policy level as well as gender and other broader equity aspects. Aliro et al. identify challenges for implementing biosecurity in smallholder pig value chains in spite of the actors understanding and accepting its importance, and propose addressing the constraints through participatory development of socially and culturally appropriate biosecurity measures.

The implementation of improved biosecurity measures is particularly relevant in areas combining a common and abundant presence of wild pigs with extensive and other outdoor farming systems in all different regions of the world, including smallholder or free-ranging pig systems in Africa (Payne et al.) or traditional pig farming systems in the Mediterranean (Gisclard et al.; Rolesu et al.) and North Macedonia (O'Hara et al.). In all cases, regardless of the geographic origin and diversity of cultural contexts, the measures required to reach a higher level of biosecurity should be flexible, adapted to the local socio-economic and cultural context and incentivised by improved trade and higher production gains.

Long-distance spread of ASF virus remains an important unsolved problem, mainly due to anthropogenic factors that can hardly be controlled. In the Samara oblast of Russia, Glazunova et al. demonstrate that outbreaks reported in backyard farms with low biosecurity were mainly related to the transport and trade of pigs and pork products from ASF-affected regions.

All three ASF impact studies in this issue relate to different parts of Asia. The study in Vietnam (Nguyen-Thi et al.) focuses on the economic effect of the ASF outbreaks and control measures across the different pig sectors, indicating that ASF might change the structure of the pig sector to a larger modern sector but that smallholder pig farmers still need to be supported. The studies in the Philippines (Cooper et al.) and Timor-Leste (Berends et al.) place a strong emphasis on the social impacts as well as economic effects on the large backyard pig farming sectors in both countries and propose participatory ASF management interventions that could mitigate the identified impacts.

Although there are very good and reliable laboratory tests for the detection of ASF (e.g., qPCR diagnostic tests), laboratory diagnosis remains a bottleneck in countries with limited laboratory capacity and financial resources. Cost-effective and alternative methods suitable for field use are therefore desirable, especially for areas with suboptimal laboratory infrastructure. The loop-mediated isothermal amplification test (LAMP) has been shown in Timor-Leste to be a robust, highly specific and sensitive diagnostic test for ASF, suitable for use in the field and in areas with limited laboratory capacity (Phillips et al.).

The article by Busch et al. argues that in order to improve ASF control, a disease-specific legal framework based on the latest scientific evidence is needed. It compares the legal basis for ASF control in a number of pig-producing regions globally, considering diverse production systems while considering current scientific evidence in relation to ASF spread and control. It is specifically emphasized that blanket policies, which do not take into account disease-relevant characteristics of a biological agent or the specifics under which the host species are kept, can hamper disease control efforts and even be counter-productive.

In summary, the results of these studies provide new and relevant insights into the challenges facing prevention and control of ASF in an array of smallholder pig farming settings, with and without wildlife involvement, and its impacts on the farmers and pig value chain actors. Based on their findings, the researchers offer constructive proposals for more participatory, inclusive and context-adapted approaches to prevention and control of ASF.

## Author Contributions

M-LP wrote the introduction and conclusion. All authors contributed to the central part relating to the individual articles and reviewed and approved the submitted version.

## Conflict of Interest

RA was employed by Kyeema Foundation. The remaining authors declare that the research was conducted in the absence of any commercial or financial relationships that could be construed as a potential conflict of interest. The handling editor declared a past collaboration with the FJ.

## Publisher's Note

All claims expressed in this article are solely those of the authors and do not necessarily represent those of their affiliated organizations, or those of the publisher, the editors and the reviewers. Any product that may be evaluated in this article, or claim that may be made by its manufacturer, is not guaranteed or endorsed by the publisher.

## References

[1] Penrith M-L. Current status of African swine fever. CABI Agric Biosci. (2020) 1:11. 10.1186/s43170-020-00011-w31538406

[2] Food Agriculture Organization of the United Nations. Update on the ASF Situation in Asia and the Pacific. (2022). Available online at https://www.fao.org/ag/againfo/programmes/en/empres/ASF/situation_update.html (accessed February 6, 2022).

[3] Food Agriculture Organization of the United Nations. African Swine Fever. FAO Regional Office for Latin America and the Caribbean. Available online at: https://www.fao.org/americas/priorities/african-swine-fever/en/ (accessed February 6, 2022).

[4] Chenais E Boqvist S Emanuelson U von Brömssen C Ouma E Aliro T. Quantitative assessment of the social and economic impact of African swine fever outbreaks in northern Uganda. Prev Vet Med. (2017) 144:134–48. 10.1016/j.prevetmed.2017.06.002 28716195

[5] Dione MM Akol J Roesel K Kungu J Ouma EA Wieland B. Risk factors for African swine fever in smallholder pig production systems in Uganda. Transbound Emerg Dis. (2017) 64:872–82. 10.1111/tbed.12542 26662861

[6] Penrith M-L Bastos A Chenais E. With or without a vaccine—complementary and alternative approaches to managing African swine fever in resource-constrained smallholder settings. Vaccines. (2021) 9:116. 10.3390/vaccines9020116 33540948 PMC7913123

